# Glucometer accuracy and implications for clinical studies

**DOI:** 10.1186/cc10780

**Published:** 2012-03-20

**Authors:** AJ Le Compte, CG Pretty, GM Shaw, JG Chase

**Affiliations:** 1 University of Canterbury, Christchurch, New Zealand; 2 Christchurch Hospital, Christchurch, New Zealand

## Introduction

Elucidating links between glycemic control and clinical outcome requires reliable discrimination between groups with different target blood glucose (BG) cut-offs. Point-of-care glucometers are commonly used, but lower accuracy means BG errors will impact classification and thus outcome analyses. This study reanalyses a BG control trial with an error model of a typical glucometer to assess the impact of sensor errors on interpretation of trial results.

## Methods

BG profiles from 301 patients (stay >24 hours) from the SPRINT trial with BG measurements (*n *= 25,000) using the Arkray SuperGlucocard II GT-1630. A model of sensor bias and variance (CV 2.7 to 3.5%, regression: *y *= 3.92 + 0.97*x*) was used to estimate possible 'true' BG profiles from measured BG and repeated 100 times for each patient. The defined cut-off for 'good' control for a patient was ≥70% of BG in 72 to 126 mg/dl (cTIB ≥0.7), and 'poor' as <70% (cTIB <0.7), based on original observed clinical BG. The number of true BG profiles that resulted in misclassification between 'good' and 'poor' control for a patient was recorded over all Monte-Carlo runs. The maximum change in true and observed BG mean and standard deviation were used to evaluate potential worst-case scenarios.

## Results

Good control was clinically measured in 76% of patients (24% with cTIB <0.7). Of these, 83% of 'good' and 64% of 'poor' control would never be misclassified over all 100 runs due to sensor error. A total of 91% (good) and 87.5% (poor) could be misclassified 10% of the time. Patients with cTIB near 0.7 were more likely to be misclassified when accounting for glucometer error. Hence, a deadband around the cut-off would reduce this misclassification. If 'good' cut-off was cTIB ≥0.5 (95% of clinical patients) then 100% correct classification was 97% for good control patients, but fell to 40% of poor control patients. The median largest difference in observed and true mean BG across patients was -54 mg/dl (90th percentile: -21 mg/dl) and the standard deviation was 3.2 mg/dl (90th percentile: 1.8 mg/dl).

## Conclusion

Glucometers can distinguish between patients that received good and poor BG control but risk of misclassification rises for patients nearer cut-offs. Reliable classification to associate with outcomes relies on the control protocol and cut-off choice to achieve sufficient separation between groups so that device errors do not result in significant misclassification confounding the results. A deadband around cut-off values to eliminate patients at high risk of misclassification may be required.

